# Insurance Types and All-Cause Mortality in Korean Cancer Patients: A Nationwide Population-Based Cohort Study

**DOI:** 10.3390/jpm14080861

**Published:** 2024-08-14

**Authors:** Jinyoung Shin, Yoon-Jong Bae, Hee-Taik Kang

**Affiliations:** 1 Department of Family Medicine, Konkuk University Medical Center, Konkuk University School of Medicine, Seoul 05030, Republic of Korea; jyshin@kuh.ac.kr; 2 Department of Data Science, Hanmi Pharm. Co., Ltd., Seoul 05545, Republic of Korea; yoonjong.bae@hanmi.co.kr; 3 Department of Family Medicine, Severance Hospital, Yonsei University College of Medicine, Seoul 03722, Republic of Korea

**Keywords:** neoplasms, malignant, mortality, economic status, healthcare disparities, insurance

## Abstract

Background: Economic deprivation is expected to influence cancer mortality due to its impact on screening and treatment options, as well as healthy lifestyle. However, the relationship between insurance type, premiums, and mortality rates remains unclear. This study investigated the relationship between insurance type and mortality in patients with newly diagnosed cancer using data from the Korean National Health Insurance Database. Methods: this retrospective cohort study included 111,941 cancer patients diagnosed between 1 January 2007 and 31 December 2008, with a median follow-up period of 13.41 years. The insurance types were categorized as regional and workplace subscribers and income-based insurance premiums were divided into tertiles (T1, T2, and T3). Results: Cox proportional hazards regression analysis adjusted for age, lifestyle factors, health metrics, and comorbidities showed workplace subscribers (*n* = 76,944) had a lower all-cause mortality hazard ratio (HR) (95% confidence interval [CI]: 0.940 [0.919–0.961]) compared to regional subscribers (*n* = 34,997). Higher income tertiles (T2, T3) were associated with lower mortality compared to the T1 group, notably in male regional and workplace subscribers, and female regional subscribers. Conclusion: The study identified that insurance types and premiums significantly influence mortality in cancer patients, highlighting the necessity for individualized insurance policies for cancer patients.

## 1. Introduction

Malignant neoplasms are the leading cause of death in South Korea, with a rate of 162.7 per 100,000 persons, followed by cardiovascular (65.8 per 100,000 persons), COVID-19 (61.0), pneumonia (52.1), and cerebrovascular diseases (49.6) in 2022 [1]. Given the global increase in the burden of cancer, including in the United States [2,3], the Korean government initiated multiple cancer prevention programs, including the National Cancer Screening Programs (NCSPs) for stomach, liver, colorectal, breast, and cervical cancers, in 1999 [4]. Participation in NCSPs increased from 12.7% in 2002 to 55.6% in 2019, while five-year relative survival rates improved from 42.9% in 1993–1995 to 70.7% in 2015–2019 [4,5,6].

According to a WHO report, the percentage of the global population that could allocate more than 10% of total household income for health increased from 12.65% in 2015 to 13.46% in 2019 [7]. However, more than 76% still face difficulties in covering medical expenses due to illness, making socioeconomic status a significant indicator of health. Socioeconomic status plays a significant role in access to health services, leading to disparities in health care [8,9,10]. Economically disadvantaged individuals engage in unhealthy behaviors, live in poor conditions, such as air pollution and low food security, and are less likely to receive early disease diagnoses or frequent medical services [11,12,13]. Moreover, manual laborers face a higher risk of mortality than non-manual laborers because of adverse work conditions and poor health habits [14]. While some studies have shown an inverse relationship between socioeconomic status and mortality in cancer [15,16,17], this has been limited to a few cancer types without considering the impact of different insurance coverage or insurance types. The National Health Insurance Service (NHIS) in South Korea covers 95% of all medical fees for cancer diagnosis and treatment for the first five years for the entire population, regardless of socioeconomic status or cancer types [18]. This suggests that socioeconomic deprivation may not significantly affect mortality rates in South Korea’s healthcare system [19]. However, few studies have investigated the association between insurance type, premium levels, and all-cause mortality.

We hypothesized that economic deprivation is an important risk factor for mortality in patients with newly diagnosed cancer because economic status could influence cancer screening and treatment options, in addition to a healthy lifestyle (including diet and behaviors) and environmental exposure to toxins or carcinogens. This study aimed to explore the association between insurance type and all-cause mortality in newly diagnosed cancer patients using the Korean National Health Insurance Database (NHID).

## 2. Materials and Methods

### 2.1. Study Design and Data Sources

A retrospective cohort study was conducted using the NHID database to assess the relationship between insurance type and all-cause mortality in Korean adults. The NHID includes demographic and medical data for the entire population (97.2% of which is insured by the NHIS and 2.8% of which is covered by the Medical Aid Program), including insurance information, hospital admissions, and medical records.

### 2.2. Study Subjects

**Figure 1:**
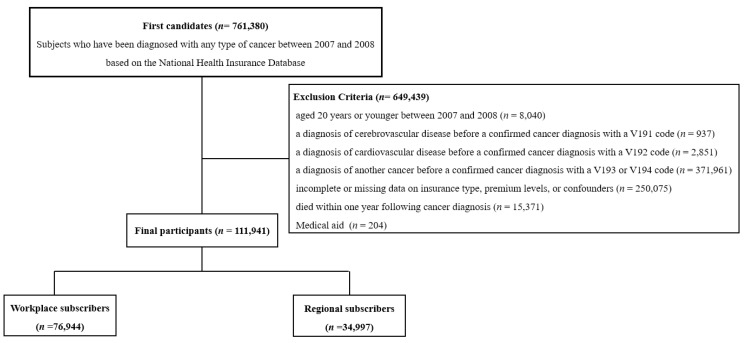
Flow chart of the study population.

A flowchart of the inclusion and exclusion criteria is shown in Figure 1. A total of 761,380 individuals diagnosed with malignant neoplasms between 2007 and 2008 were enrolled in this study. Exclusions were made for individuals under 20 years of age during the study period (*n* = 8040), those with prior diagnoses of cerebrovascular disease (*n* = 937, V191 code) or cardiovascular disease (*n* = 2851, a V192 code), those with another cancer diagnosis (*n* = 371,961, a V193 or V194 code), those with incomplete data on insurance type, premium levels, or confounders (*n* = 250,075), those who died within one year after diagnosis (*n* = 15,371), and those receiving Medical Aid (*n* = 204) due to the differing payment system from the National Health Insurance (NHI). After these exclusions, 111,941 participants (55,563 men and 56,378 women) were included in the analysis.

### 2.3. Definitions of Insurance Types, Malignancy, and Confounders

In South Korea, NHI categorizes subscribers into two main groups based on their occupation: workplace and regional groups. Additionally, economically disadvantaged individuals are classified under medical aid, which is designed to provide healthcare benefits to those who need financial assistance [20]. Workplace and regional groups are divided into the 0–20th level of household income to determine insurance premiums. In this study, individuals within each group were further categorized into tertiles (T1, 0–6th; T2, 7th–13th; and T3, 14th–20th), allowing for a more detailed analysis of the impact of income level on various health outcomes within these insurance categories.

The NHIS health screening cohort provided self-administered medical histories, anthropometric measurements, health-related behaviors, and laboratory tests. Patients with malignant neoplasms received diagnosis codes V193 and V194, sorted by the International Classification of Diseases, 10th revision (ICD-10) codes C00–C97, D00–D09, D32–D33, and D37–D48. The V194 code is for registered cancer patients who receive home care services. The Korean NHIS strictly monitors these V193 and 194 codes as special diseases, supplying benefits for five years after the initial cancer diagnosis. The primary endpoint of this study was the comparison of all-cause mortality by insurance type after enrollment (2007–2008). Death was recorded when a death certificate was issued, regardless of cause.

The age at cancer diagnosis was calculated based on the participants’ birth year and year of diagnosis. Body mass index (BMI) was calculated as weight divided by the square of height (kg/m^2^). Smoking status was classified as never smoked, former smokers, or current smokers. Alcohol consumption was classified as mild less than one drink per week; moderate, 1–3 drinks per week; and heavy, more than four drinks per week. Physical activity was classified as rare, less than once a week; moderate, 1–3 times a week; and regular, more than four times a week. Blood pressure was measured during health checkups. Laboratory analysis of the venous blood was performed using samples collected after overnight fasting. We obtained the levels of fasting blood sugar, total cholesterol, alanine transaminase, and γ-glutamyl transpeptidase. A Charlson comorbidity index (CCI) score was calculated based on diagnostic codes, which were developed in 1984 and have been validated to measure comorbidities [21,22,23,24].

The research start date was the first day of cancer diagnosis using the V193 or V194 codes at baseline (2007–2008). If a patient died, the end date was the day of death recorded on the death certificate until 31 December 2021. Otherwise, the end date was the date of the last outpatient clinic visit or the last health checkup.

### 2.4. Statistical Analysis

Continuous variables are presented as the mean ± standard deviation, and categorical variables are presented as the number of subjects (percentage). Differences between groups were analyzed using the Student’s *t*-test, chi-square test, and analysis of variance, as appropriate. Kaplan–Meier survival curves were plotted, and Cox proportional hazards regression analysis was performed to adjust for various factors, including age, smoking status, alcohol consumption, physical activity, household income, BMI, blood pressure, laboratory tests (fasting blood sugar, total cholesterol, alanine transaminase, and γ-glutamyl transpeptidase levels), and CCI (except for malignancy). Statistical significance was set at *p* < 0.05. Analyses were conducted using SAS Enterprise Guide version 9.4 (SAS Inc., Cary, NC, USA) and R studio version 3.5.2. The R Project for Statistical Computing is available online: https://www.r-project.org/ (accessed on 13 October 2023).

## 3. Results

The baseline characteristics of the 111,941 patients with cancer (55,563 men and 56,378 women) are shown in Table 1. The distribution of regional and workplace insurance subscribers was 31.3% and 68.7%, respectively.

Notably, workplace subscribers were diagnosed with cancer at younger ages than regional subscribers, with average ages of 61.3 years for men and 56.1 years for women, compared to 63.6 years and 57.6 years, respectively, for workplace subscribers. Female workplace subscribers had a lower average BMI than their regional counterparts, whereas the difference in BMI between male subscribers was not statistically significant. Among both sexes, regional subscribers showed higher percentages of current smokers, heavy drinkers, regular physical activity, multiple comorbidities, and higher economic level than workplace subscribers.

Table 2 details the population characteristics based on the income-stratified insurance premiums. Among regional subscribers, the youngest average age at cancer diagnosis was observed in the highest income tertile (T3) for both sexes. The highest percentage of never smokers was found in T3 across both regional and workplace groups. Drinking habits varied, with T3 male regional subscribers having the lowest percentage of rare drinkers, while the highest percentage was observed among T3 females across both subscriber types. The percentage of regular physical activity increased from T1 to T3 in both the groups. The incidence of multiple comorbidities (CCI ≥ 2) was lowest among T3 regional subscribers of both sexes, with no significant difference in male workplace subscribers, but an increasing trend in female workplace subscribers.

**Figure 2:**
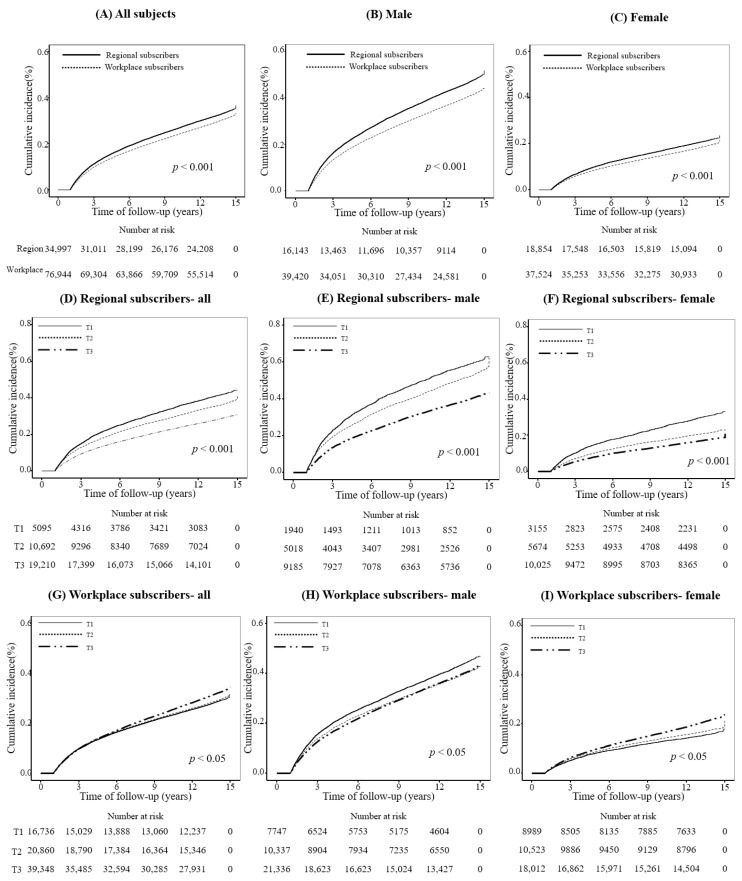
Cumulative incidence of all-cause mortality in study population (**A**–**I**).

Figure 2 shows the Kaplan–Meier survival curves, suggesting the relationship between insurance type, economic status, and all-cause mortality. Regional subscribers experienced higher all-cause mortality rates than workplace subscribers across all patient categories (*p* < 0.001, calculated using log-rank tests, Figure 2A–C). Wealthier regional subscribers (T2 and T3) had lower mortality rates in all groups (*p* < 0.001; Figure 2D–F). However, this trend was not mirrored among workplace subscribers, despite the significant differences (*p* < 0.001, Figure 2G–I).

Table 3 presents the Cox proportional hazards regression models that analyze all-cause mortality by insurance type. Workplace subscribers exhibited lower crude mortality risks compared to regional subscribers; the crude hazard ratio (HRs) (95% confidence intervals [CIs]) for all-cause mortality of workplace subscribers was 0.891 (0.872–0.911) in all patients, 0.826 (0.804–0.849) in men, and 0.880 (0.847–0.915) in women. After full adjustment for age, smoking status, alcohol consumption, physical activity, economic status, BMI, systolic blood pressure, laboratory results, and CCI, the HRs (95% CI) were 0.940 (0.919–0.961), 0.922 (0.897–0.948), and 0.925 (0.890–0.962) in all patients, men, and women, respectively.

Table 4 focuses on mortality risk according to economic status within each insurance type, showing that higher economic status correlates with lower mortality risk. This trend is consistent for both regional and workplace subscribers. Compared with T1 in regional subscribers, the HRs (95% CI) for all-cause mortality of T2 and T3 were 0.925 (0.864–0.992) and 0.730 (0.682–0.781), respectively, in males, and 0.874 (0.803–0.952) and 0.777 (0.717–0.841), respectively, in females. Compared with T1 workplace subscribers, the HRs (95% CI) of T2 and T3 workplace subscribers were 0.950 (0.907–0.994) and 0.880 (0.846–0.916), respectively, in males, and 0.986 (0.920–1.057) and 0.883 (0.830–0.940), respectively, in females.

## 4. Discussion

This study provides evidence of the association between insurance type and mortality risk in South Korea, suggesting that workplace insurance subscribers exhibit lower mortality risks than their regional counterparts, a trend consistent across sexes. Furthermore, within each insurance category, a lower economic status correlates with increased all-cause mortality for patients of both sexes newly diagnosed with cancer.

Previous studies, including those on breast cancer patients in South Korea [16] and cervical cancer patients in Bucaramanga, Colombia [25], on universal health insurance have similarly shown variations in mortality rates based on insurance type and economic status. These findings emphasize that universal health insurance does not eliminate mortality disparities influenced by socioeconomic factors and insurance type.

This investigation is unique in that it explores mortality rates within the same NHI but among different insurance types. It acknowledges the complex interplay of factors affecting mortality in cancer patients, including clinical (e.g., time since cancer diagnosis, age at cancer diagnosis, and cancer type), individual (age, sex), socioeconomic status (education, income, health insurance), lifestyle, and comorbidity factors (such as obesity, diabetes, hypertension, and dyslipidemia) [26]. Our findings underscore the significance of insurance type as a determinant of mortality even after adjusting for confounding factors.

We observed that regional subscribers, who often receive diagnoses at older ages and may have less healthy lifestyle habits and more comorbid conditions, exhibit higher mortality rates [27]. This suggests that workplace subscribers are likely to benefit from more regular cancer screenings and broader healthcare access. The study also highlights how economic status within the same insurance category affects mortality, suggesting that socioeconomic factors restrict early cancer detection and treatment options [15,28]. Furthermore, female workplace subscribers showed an increasing trend in mortality with rising income levels without adjusting for confounding factors. However, after adjusting for confounding factors, an increase in income level was significantly associated with a decrease in mortality. This suggests that other influencing variables could control for the association between premium income levels and mortality. Even after cancer diagnosis, individuals with a low economic status may have fewer treatment options and find it difficult to find proper treatment. Patients may experience perceived or realized financial obstacles when making treatment decisions in a universal healthcare system [29]. Poor management of comorbidities, in addition to cancer, can increase the risk of mortality. Additionally, patients may develop unhealthy lifestyle habits or have an increased exposure to carcinogens. This is exacerbated in a universal healthcare system, where financial barriers, despite coverage, can impede access to comprehensive care and lead to poorer outcomes.

The advantages of this study include the use of real-world data encompassing the entire Korean population and a methodology that minimizes the risk of diagnostic misclassification through the use of specific diagnostic codes (V codes), of which 95% of medical expenses are largely covered by insurance. These patients pay less out-of-pocket (only 5%) for all medical costs than do patients with other general diseases. Thus, the V193 and V194 codes better reflect real-world diagnoses of malignant neoplasms than the C codes among the ICD-10 codes. The extensive follow-up period (median years [interquartile range]: 13.41, [4.10]) enriched the analysis of economic status and mortality in cancer patients.

However, this study has limitations, including the inability to account for cancer type, stage, and treatment, as well as the absence of data on health check-up frequencies and additional socioeconomic variables such as education levels [28]. Salaries, wages, investment income, and assets should be considered as the factors that determine income levels. However, we were unable to obtain these data because they were not covered by the NHIS. The calculation methods for premiums also vary between workers and regional subscribers who have different average incomes across strata. Income disparities may not be consistent across insurance types. These limitations highlight the need for further research to deepen our understanding of targeted interventions. This study utilized V codes to classify cancer patients, thereby reducing false-positive diagnoses accurately. However, this approach led to the exclusion of many participants. Therefore, it is necessary to consider this when interpreting the results. Lastly, although it is common practice to assume a normal distribution or to omit normality testing in large-scale population data, this study did not show the normality with a statistical method.

## 5. Conclusions

Insurance type is an independent factor for mortality, with workplace subscribers and individuals with a higher economic status experiencing better outcomes. These findings call for further research into the specific mechanisms driving these disparities, and for policy interventions aimed at reducing mortality among cancer patients with lower socioeconomic status. Allocating additional healthcare resources to the economically disadvantaged could mitigate these disparities, moving towards more equitable health outcomes for all citizens.

## Figures and Tables

**Table 1:** Baseline characteristics according to insurance type by sex (*n* = 111,941).

	Male		*p*-Value	Female		*p*-Value
	Regional Subscribers	Workplace Subscribers		Regional Subscribers	Workplace Subscribers	
Number	16,143	39,420		18,854	37,524	
Age at cancer diagnosis, year	63.6 ± 10.4	61.3 ± 12.2	<0.001	57.6 ± 10.6	56.1 ± 12.5	<0.001
Body mass index, kg/m^2^	23.6 ± 3.0	23.6 ± 3.0	0.066	24.0 ± 3.3	23.6 ± 3.2	<0.001
Smoking status, *N* (%)			<0.001			<0.001
Never	8269 (51.2)	20,855 (52.9)		17,935 (95.1)	36,534 (97.4)	
Former	3007 (18.6)	7843 (19.9)		263 (1.4)	421 (1.1)	
Current	4867 (30.1)	10,722 (27.2)		656 (3.5)	569 (1.5)	
Alcohol consumption, *N* (%)			<0.001			<0.001
Mild	8451 (52.3)	20,224 (51.3)		15,721 (83.4)	31,654 (84.4)	
Moderate	4461 (27.6)	13,059 (33.1)		2740 (14.5)	5502 (14.7)	
Heavy	3231 (20.0)	6137 (15.6)		393 (2.1)	368 (0.98)	
Physical activity, *N* (%)			<0.001			<0.001
Rare	8292 (51.4)	18,001 (45.7)		10,436 (55.4)	20,220 (53.9)	
Moderate	5403 (33.5)	16,027 (40.7)		6144 (32.6)	13,190 (35.2)	
Regular	2448 (15.2)	5392 (13.7)		2274 (12.1)	4114 (11.0)	
Charlson comorbidity index, *N* (%)			<0.001			0.007
0	3154 (19.5)	8898 (22.6)		5263 (27.9)	10,870 (29.0)	
1	6314 (39.1)	15,704 (39.8)		7853 (41.7)	15,645 (41.7)	
≥2	6675 (41.3)	14.818 (37.6)		5738 (30.4)	11,009 (29.3)	
Household income level, *N* (%)			<0.001			<0.001
Lowest	1940 (12.0)	7747 (19.7)		3155 (16.7)	10,870 (29.0)	
Middle	5018 (31.1)	10,337 (26.2)		5674 (30.1)	15,645 (41.7)	
Highest	9185 (56.9)	21,336 (54.1)		10,025 (53.2)	11,009 (29.3)	

Data are presented as *N* (%) for categorical variables or mean ± standard deviation (SD) for continuous variables.

**Table 2:** Clinical variables according to economic status by insurance type.

Male	Regional Subscribers (*n* = 16,143)			*p*-Value	Workplace Subscribers (*n* = 39,420)			*p*-Value
	T1	T2	T3		T1	T2	T3	
Number	1940	5018	9185		7747	10,337	21,336	
Age at cancer diagnosis, year	65.9 ± 11.4	63.8 ± 11.0	63.1 ± 9.9	<0.001	62.0 ± 10.0	59.8 ± 12.0	61.8 ± 12.9	<0.001
Body mass index, kg/m^2^	23.2 ± 3.2	23.4 ± 3.1	23.8 ± 2.9	<0.001	23.6 ± 3.0	23.6 ± 3.0	23.6 ± 2.9	0.534
Smoking status, *N* (%)				<0.001				<0.001
Never	932 (48.0)	2462 (49.1)	4875 (53.1)		3915 (50.5)	5166 (50.0)	11,774 (55.2)	
Former	327 (16.9)	832 (16.6)	1848 (20.1)		1315 (17.0)	1947 (18.8)	4581 (21.5)	
Current	681 (35.1)	1724 (34.4)	2462 (26.8)		2517 (32.5)	3224 (31.2)	4981 (23.3)	
Alcohol consumption, *N* (%)				<0.001				<0.001
Mild	1074 (55.4)	2691 (53.6)	4686 (51.0)		3932 (50.8)	5126 (49.6)	11,166 (52.3)	
Moderate	494 (25.5)	1256 (25.0)	2711 (29.5)		2464 (31.8)	3392 (32.8)	7203 (33.8)	
Heavy	372 (19.2)	1071 (21.3)	1788 (19.5)		1351 (17.4)	1819 (17.6)	2967 (13.9)	
Physical activity, *N* (%)				<0.001				<0.001
Rare	1135 (58.5)	2879 (57.4)	4278 (46.6)		3791 (48.9)	5005 (48.4)	9205 (43.1)	
Moderate	544 (28.0)	1462 (29.1)	3397 (37.0)		2983 (38.5)	3985 (38.6)	9059 (42.5)	
Regular	261 (13.5)	677 (13.5)	1510 (16.4)		973 (12.6)	1347 (13.0)	3072 (14.4)	
Charlson comorbidity index, *N* (%)				<0.001				0.074
0	368 (19.0)	925 (18.4)	1861 (20.3)		1687 (21.8)	2288 (221)	4923 (23.1)	
1	719 (37.1)	1927 (38.4)	3668 (39.9)		3093 (39.9)	4183 (40.5)	8428 (39.5)	
≥ 2	853 (44.0)	2166 (43.2)	3656 (39.8)		2967 (38.3)	3866 (37.4)	7985 (37.4)	
**Female**	**Regional Subscribers (*n* = 18,854)**			***p*-Value**	**Workplace Subscribers (*n* = 37,524)**			***p*-Value**
	**T1**	**T2**	**T3**		**T1**	**T2**	**T3**	
Number	3155	5674	10,025		8989	10,523	18,012	
Age at cancer diagnosis, year	60.5 ± 12.4	57.2 ± 10.7	56.9 ± 9.7	<0.001	53.6 ± 11.1	54.1 ± 12.6	58.6 ± 12.6	<0.001
Body mass index, kg/m^2^	23.9 ± 3.4	24.0 ± 3.4	24.0 ± 3.2	0.573	23.7 ± 3.2	23.7 ± 3.3	24.5 ± 3.2	<0.001
Smoking status, *N* (%)				<0.001				<0.001
Never	2855 (90.5)	5345 (94.2)	9735 (97.1)		8710 (96.9)	10,195 (96.9)	17,629 (97.9)	
Former	68 (2.2)	91 (1.6)	104 (1.0)		111 (1.2)	140 (1.3)	170 (0.9)	
Current	232 (7.4)	238 (4.2)	186 (1.9)		168 (1.9)	188 (1.8)	213 (1.2)	
Alcohol consumption, *N* (%)				<0.001				<0.001
Mild	2599 (82.4)	4688 (82.6)	8434 (84.1)		7255 (80.7)	8672 (82.4)	15,727 (87.3)	
Moderate	460 (14.6)	851 (15.0)	1429 (14.3)		1619 (18.0)	1730 (16.4)	2153 (12.0)	
Heavy	96 (3.0)	135 (2.4)	1629 (1.6)		115 (1.3)	121 (1.2)	132 (0.7)	
Physical activity, *N* (%)				<0.001				<0.001
Rare	1986 (62.9)	3358 (59.2)	5092 (50.8)		5063 (56.3)	5913 (56.2)	9244 (51.3)	
Moderate	846 (26.8)	1649 (29.1)	3649 (36.4)		3030 (33.7)	3537 (33.6)	6623 (36.8)	
Regular	323 (10.2)	667 (11.8)	1284 (12.8)		896 (10.0)	1073 (10.2)	2145 (11.9)	
Charlson comorbidity index, *N* (%)				0.002				<0.001
0	828 (26.4)	1539 (27.1)	2896 (28.9)		2729 (30.4)	3162 (30.0)	4979 (27.6)	
1	1296 (41.1)	2375 (41.9)	4182 (41.7)		3845 (42.8)	4363 (41.5)	7437 (41.3)	
≥2	1031 (32.7)	1760 (31.0)	2947 (29.4)		2415 (26.9)	2998 (28.5)	5596 (31.1)	

Data are presented as *N* (%) or mean ± standard deviation (SD). *p*-values were obtained using analysis of variance (ANOVA) or chi-square tests.

**Table 3:** Cox proportional hazards regression model for all-cause mortality according to insurance types in Korean cancer patients.

	All		Male		Female	
	Regional Subscribers	Workplace Subscribers	Regional Subscribers	Workplace Subscribers	Regional Subscribers	Workplace Subscribers
Model 1	1	0.891 (0.872–0.911)	1	0.826 (0.804–0.849)	1	0.880 (0.847–0.915)
Model 2	1	0.946 (0.925–0.967)	1	0.919 (0.894–0.945)	1	0.929 (0.893–0.966)
Model 3	1	0.940 (0.919–0.961)	1	0.922 (0.897–0.948)	1	0.925 (0.890–0.962)

Model 1: univariate; Model 2: adjusted for age (sex in case of all population), smoking status, alcohol drinking, physical activity, and economic status; Model 3: body mass index, systolic blood pressure, laboratory results (fasting blood sugar, total cholesterol, alanine transaminase, γ-glutamyl transpeptidase), and Charlson comorbidity index (except for malignancy) in addition to variables of Model 2.

**Table 4:** Cox proportional hazards regression model for all-cause mortality according to economic status in Korean cancer patients.

		Regional Subscribers			Workplace Subscribers		
		T1	T2	T3	T1	T2	T3
Male	Model 1	1	0.835 (0.770–0.895)	0.572 (0.536–0.611)	1	0.874 (0.830–0.915)	0.881 (0.847–0.917)
	Model 2	1	0.925 (0.863–0.991)	0.700 (0.654–0.748)	1	0.950 (0.908–0.994)	0.871 (0.867–0.907)
	Model 3	1	0.925 (0.864–0.992)	0.730 (0.682–0.781)	1	0.950 (0.907–0.994)	0.880 (0.846–0.916)
Female	Model 1	1	0.655 (0.603–0.713)	0.529 (0.489–0.571)	1	1.098 (1.025–1.177)	1.387 (1.306–1.474)
	Model 2	1	0.876 (0.805–0.953)	0.774 (0.715–0.839)	1	0.993 (0.926–1.064)	0.898 (0.844–0.956)
	Model 3	1	0.874 (0.803–0.952)	0.777 (0.717–0.841)	1	0.986 (0.920–1.057)	0.883 (0.830–0.940)

Model 1: univariate; Model 2: adjusted for age, smoking status, alcohol drinking, physical activity, and economic status; Model 3: body mass index, systolic blood pressure, laboratory results (fasting blood sugar, total cholesterol, alanine transaminase, γ-glutamyl transpeptidase), and Charlson comorbidity index (except for malignancy) in addition to variables of Model 2.

## Data Availability

The data supporting this article are accessible from the National Health Insurance Service’s Open Data Portal (https://opendata.hira.or.kr/home.do).
